# Survival and mortality profile among people living with HIV in a cohort in the Northeastern region of Brazil

**DOI:** 10.1590/S1678-9946202466023

**Published:** 2024-04-19

**Authors:** Kaliene Maria Estevão Leite, Kledoaldo Oliveira Lima, Ricardo Arraes de Alencar Ximenes, Maria de Fatima Militão de Albuquerque, Demócrito de Barros Miranda-Filho, Emmanuelle Tenório Albuquerque Madruga Godoi, Ulisses Ramos Montarroyos, Heloísa Ramos Lacerda

**Affiliations:** 1 Universidade Federal de Pernambuco, Pós-Graduação em Medicina Tropical, Recife, Pernambuco, Brazil; 2 Universidade Federal de Pernambuco, Hospital das Clínicas, Recife, Pernambuco, Brazil; 3 European Virus Bioinformatics Center, Jena, Germany; 4 Instituto de Medicina Integral Prof. Fernando Figueira, Faculdade Pernambucana de Saúde, Recife, Pernambuco, Brazil; 5 Universidade de Pernambuco, Pós-Graduação em Ciências da Saúde, Recife, Pernambuco, Brazil; 6 Fundação Oswaldo Cruz, Instituto de Pesquisa Aggeu Magalhães, Recife, Pernambuco, Brazil

**Keywords:** HIV, Mortality, Causes of death, AIDS, Survival

## Abstract

Conditions related to the acquired immune deficiency syndrome (AIDS) are still a significant cause of morbidity and mortality among people living with HIV (PLHIV). Longer survival in this population were reported to increase the risk of developing noncommunicable chronic diseases (NCDs). This study aimed to estimate the survival and causes of death according to age group and sex among PLHIV monitored at two referral centers in the Northeastern Brazil. This is a prospective, retrospective cohort with death records from 2007 to 2018, based on a database that registers causes of death using the International Classification of Disease (ICD-10), which were subsequently coded following the Coding Causes of Death in HIV (CoDe). A total of 2,359 PLHIV participated in the study, with 63.2% being men, with a follow-up period of 13.9 years. Annual mortality rate was 1.46 deaths per 100 PLHIV (95% CI: 1.33 – 1.60) with a frequency of 20.9%. Risk of death for men increased by 49% when compared to women, and the risk of death in PLHIV increased by 51% among those aged 50 years and over at the time of diagnosis. It was observed that 73.5% accounted for AIDS-related deaths, 6.9% for non-AIDS defining cancer, 6.3% for external causes, and 3.2% for cardiovascular diseases. Among the youngest, 97.2% presented an AIDS-related cause of death. Highest frequency of deaths from neoplasms was among women and from external causes among men. There is a need for health services to implement strategies ensuring greater adherence to treatment, especially among men and young people. Moreover, screening for chronic diseases and cancer is essential, including the establishment of easily accessible multidisciplinary care centers that can identify and address habits such as illicit drug use and alcoholism, which are associated with violent deaths.

## INTRODUCTION

The implementation of human immunodeficiency virus (HIV) treatment on a global scale and the facilitated access to antiretroviral therapy (ART) has resulted in a significant reduction in the mortality of people living with HIV (PLHIV). It is estimated that 16.6 million AIDS-related deaths were prevented over the last two decades^1^, with life expectancy now approaching that seen in the general population^2^. In Brazil, from 2010 to 2020, a decrease of 29.9% was observed in the standardized mortality rate among PLHIV, which dropped from 5.7 to 4.0 deaths per 100,000 inhabitants^3^.

Studies conducted in Europe, the USA, and Australia show that AIDS-related conditions continue to be a significant cause of morbidity and mortality among PLHIV, but early diagnosis of infection, the use of ART, and the consequent increase in survival rates has brought about a higher age group in this population^4^. Thus, an increase in the risk of developing noncommunicable chronic diseases (NCDs) has been noted along with a decrease in the incidence of events associated with AIDS^5^. The risk of myocardial infarction, end-stage renal disease, and non-AIDS-defining malignancies are higher in PLHIV compared with people without the virus, and deaths caused by neoplasms and cardiovascular diseases have been shown to be high^6^. In Brazil, non-HIV-related causes of death among PLHIV, such as liver and cardiovascular diseases and neoplasms, demonstrated an annual growth trend from 2000 to 2010 compared to the period before 1999^7,8^.

A study conducted by Alves *at al*.^9^ used the Coding Causes of Death in HIV (CoDe) to classify the death profile in Northeastern Brazil from 2007 to 2012, observed that although most deaths were related to immunodeficiency (73.6%), those unrelated to AIDS, such as cardiovascular diseases, neoplasms, and external causes, appeared among the causes of death. Thus, it became relevant to monitor this population for a longer period to assess the profile of causes of death over time. Monitoring the causes of death is important to guide public health programs aimed at preventing and monitoring the health of the population living with HIV, with a view to prolonging their survival. Thus, this study aimed to estimate the survival time and describe the causes of death in PLHIV monitored at two referral services in Pernambuco State, in Northeastern Brazil, from 2007 to 2018.

## MATERIALS AND METHODS

### Study population

This is a prospective cohort study that considered patient follow-up from their inclusion in the research until the date of death. It also presents a retrospective analysis, considering the data collection before the patient’s inclusion in the research such as diagnosis of infection and initiation of antiretroviral therapy, which were obtained via medical records. It was based on a secondary database containing the causes of death following the International Classification of Diseases (ICD-10). This database presents survival rates and causes of death according to age group and death in a cohort of 2,359 PLHIV followed at two reference centers in Pernambuco State, Northeastern Brazil (AIDS-PE Cohort). The study included individuals of both sexes, aged ≥ 18 years, registered in the HIV/AIDS monitoring, and undergoing treatment program at one of the hospitals where the study was conducted. The follow-up of the cohort continued until December 2018. Both reference centers offer patient care from multiple specialties, in addition to laboratory and imaging diagnosis.

### Data collection

From 2007 to 2010, patients were included in the study. They signed an informed consent form and answered a standardized questionnaire covering general information. Data were collected from medical records regarding the dates when infection was diagnosed and antiretroviral therapy was initiated. Although the inclusion period for patients was from 2007 to 2010, the follow-up period for the occurrence of death from the HIV diagnosis date until December 2018, whereby the longest follow-up period was from August 1986 to December 2018. The study was based on a secondary database containing the causes of death registered into the ICD-10, retrieved from the database of the Mortality Information System in Pernambuco State Health Department-SIM/PE. The database provided information on the main cause of death, as well as all information on variables such as age, sex, and ART treatment.

### Categorizing the causes of death

The detected deaths were then coded using the CoDe protocol^10^, which standardizes the classification of causes of death in PLHIV using death certificate data and clinical markers. To appropriately use the CoDe in the study, the methodology outlined in Alves *et al*.^9^ was followed, in which the CoDe codes and the respective instructions were translated by specialists into Brazilian Portuguese. The ICD-10 death classification codes were inserted into the corresponding categories of the CoDe, and adapted to form 11 groups: Group 1 – AIDS, which included the categories AIDS-ongoing-active disease and AIDS-Infection; Group 2 – Cancer AIDS, Group 3 – Cancer; Group 4 – External causes (accident/violent death); Group 5 – Cardiovascular diseases; Group 6 – Nervous system diseases; Group 7 – Genitourinary disorders; Group 8 – Respiratory system diseases; Group 9 – Liver diseases; Group 10 – Digestive system diseases; and Group 11 – Other causes.

### Statistical analysis

A descriptive analysis of the characteristics of PLHIV was performed by analyzing the age and sex profiles, then associating these characteristics to the time of diagnosis and time of death. The distribution by cause of death was described, which presented the absolute frequency and percentage of the variables sex and ART treatment. The association of these variables with the cause of death, together with the median and interquartile range for age, was assessed using the Kruskal-Wallis test. The dependent variable of the study was the time until death, with an estimated incidence density of 100 person-years, with a 95% confidence interval. The probability of survival was estimated using the Kaplan-Meier method and was represented by the cumulative survival curve. Survival functions per sex and age group were compared using the Log-Rank test. The hazard ratio (HR) was the measure of association adopted for the study and was estimated using the Cox proportional-hazards model (Cox regression). The Schoenfeld residual was used for proportionality test, and proportionality was confirmed in the study associations, meeting the assumption of the model. Data analysis was conducted in Stata 14 program and the significance adopted in the study was 5% (p<0.05).

### Ethical considerations

The research was approved by the Research Ethics Committees at the Universidade Federal de Pernambuco (CAAE Nº 80347517.2.0000.5208) and Universidade de Pernambuco (CAAE Nº 80347517.2.3001.5192). Consent was granted by the Pernambuco State Health Department (SES/PE) to access the database of the Mortality Information System (SIM) (Process Nº 2300000157.000099/2021-77).

## RESULTS

A total of 2,359 PLHIV participated in the study, of whom 63.2% were male. The median age of the participants at the time of HIV diagnosis was 33.6 years. In the age group distribution, 31.7% were aged from 20 to 29 years, 37.2% from 30 to 39 years, 4.3% were aged under 20 years, and only 1% were aged 60 years and over. At the time of data collection, 85.7% of participants were undergoing ART, and the time between diagnosis and initiating ART was 6.3 months. The frequency of death among surveyed patients was 20.9%, in which the median age at death was 44 years and the interquartile range from 36 to 51 years (Table 1).

**Table 1:** Characteristics of the cohort of people living with HIV/AIDS.

Characteristic	Statistics
**Number of participants**	2,359
**Sex**	
Female	868 (36.8%)
Male	1,491 (63.2%)
**Age at HIV diagnosis (in years)**	
Median (P_25_ – P_75_)	33.6 (27.2 – 40.5)
**Age group at the time of HIV diagnosis (in years)**	
Under 20	102 (4.3%)
20 to 29	749 (31.7%)
30 to 39	877 (37.2%)
40 to 49	471 (20.0%)
50 to 59	136 (5.8%)
60 and over	24 (1.0%)
**Using ART on the interview date**	
Yes	2,023 (85.7%)
No	336 (14.2%)
**Time between diagnosis and initiating ART (in months)**	
Median (P_25_ – P_75_)	6.3 (2.1 – 27.7)
**Death**	
Yes	492 (20.9%)
No	1,867 (79.1%)
**Age at death (in years)**	
Median (P_25_ – P_75_)	44 (36 – 51)

**Figure 1:**
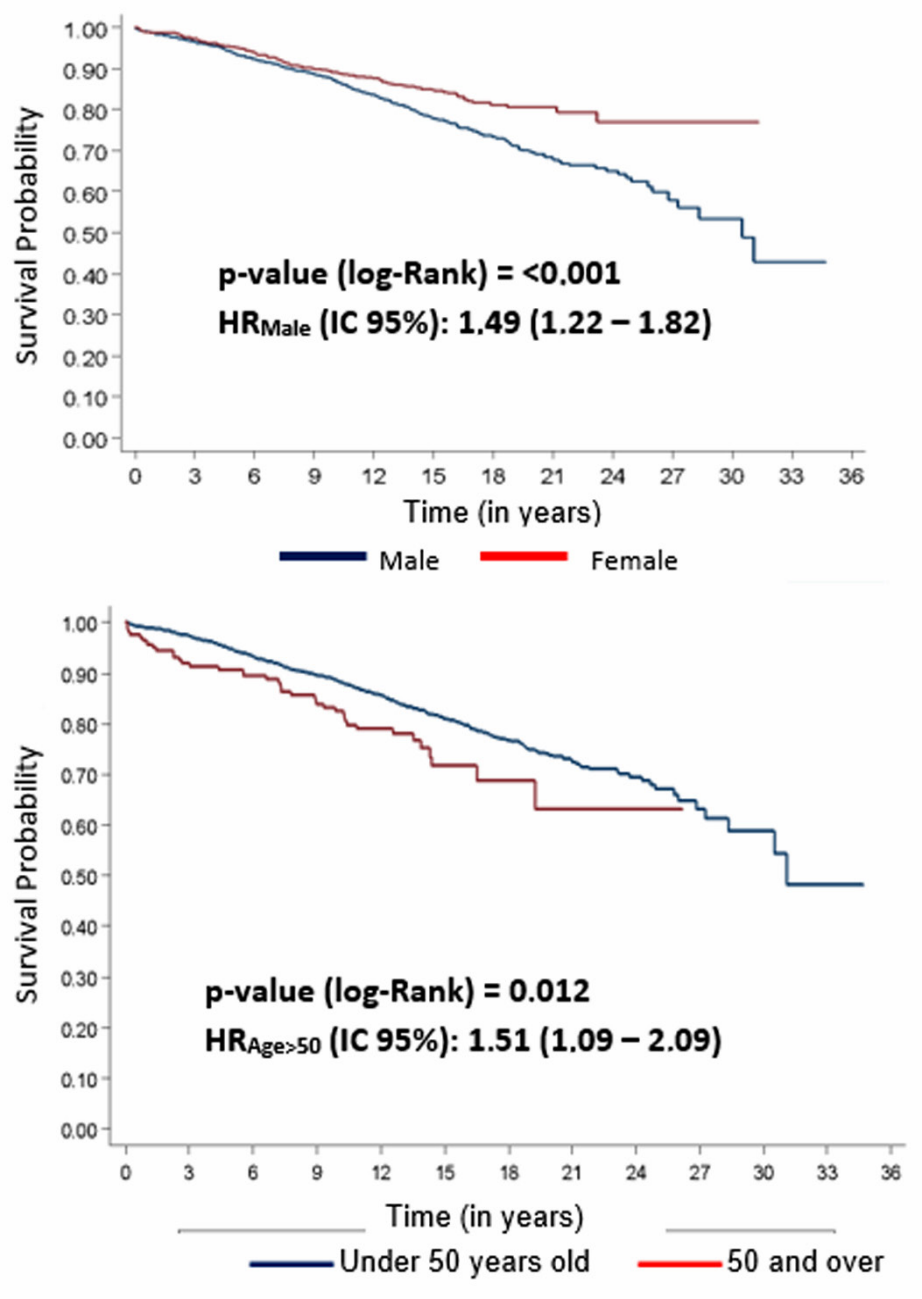
Survival of people living with HIV according to the Kaplan-Meier method by sex and age group at the time the infection.

For the survival analysis, the cohort follow-up time was 13.9 years, with an annual mortality rate of 1.46 deaths per 100 PLHIV (95% CI: 1.33 – 1.60). The probability of death during the first year was 1.3%, while by the fifth year this percentage increased to 5.6% and by up to 20 years the probability was 26.7% (Table 2). At the time when the infection was diagnosed, a statistically significant difference was observed between the survival functions when comparing sexes, with a 49% higher risk of death for men when compared to women. In terms of age, a difference was observed between the survival curves, which demonstrated a higher mortality rate among those aged 50 years or older at the time of HIV diagnosis, with a significant increase of 51% in the risk of death. (Figure 1).

**Table 2:** Description of time of follow-up, mortality rate, and cumulative probability of death.

Characteristic	Statistics
**Time of follow-up**	
Median (Minimum – Maximum)	13.9 years (1 month – 34.7 years)
**Mortality rate (95% CI)**	
100 people per year	1.46 (1.33 – 1.60)
**Cumulative probability of death (CI 95%)**	
Up to 1 year (n = 2,359)	1.3% (0.9 – 1.8)
Up to 2 years (n = 2,329)	1.9% (1.4 – 2.5)
Up to 5 years (n = 2,261)	5.6% (4.7 – 6.6)
Up to 10 years (n = 2,086)	12.0% (10.8 – 13.4)
Up to 15 years (n = 1,174)	19.5% (17.8 – 21.3)
Up to 20 years (n = 417)	26.7% (24.4 – 29.3)

When describing the main causes of death with the CoDe, it may be observed that 73.5% of deaths were related to AIDS, 6.9% to non-AIDS-related cancer, 6.3% to external causes (accident/violent death), and 3.3% to cardiovascular diseases (Table 3). Associating the causes of death in relation to sex, there was a higher frequency of cancer among females and a higher frequency of external causes among males. Regarding age groups, in 97.2% of the youngest participants (20–29 years old) the cause of death was AIDS-related, drawing attention to deaths from external causes in those aged from 30 to 49 years old. With an increase in the age group, the frequencies of deaths related to cancer increased, particularly in the age groups above 40 years (Table 4 and Table 5).

**Table 3:** Cause of death of people living with HIV/AIDS, adapted following the Coding Causes of Death in HIV (CoDe) classification.

Cause of death	n (%)
Group 1 – AIDS	362 (73.5%)
Group 2 – Cancer AIDS	7 (1.4%)
Group 3 – Cancer	34 (6.9%)
Group 4 – External causes (accidents/violent death)	31 (6.3%)
Group 5 – Cardiovascular diseases	16 (3.2%)
Group 6 – Nervous system diseases	4 (0.8%)
Group 7 - Genitourinary disorders	6 (1.2%)
Group 8 – Respiratory diseases	8 (1.6%)
Group 9 – Liver diseases	8 (1.6%)
Group 10 – Digestive system diseases	4 (0.8%)
Group 11 – Other causes	12 (2.4%)

**Table 4:** Distribution of the underlying cause of death in people living with HIV/AIDS according to sex and age group.

Characteristic	Male (n = 356)	Female (n = 136)	20 to 29 (n = 36)	30 to 39 (n = 159)	40 to 49 (n = 161)	50 to 59 (n = 100)	60+ (n = 36)
**Underlying cause – CoDe Database**							
AIDS - ongoing active disease	37 (10.3%)	16 (1.1%)	3 (8.3%)	21 (1.3%)	16 (9.9%)	10 (10.0%)	3 (8.3%)
Infection – AIDS	205 (57.5%)	80 (58.8%)	30 (83.3%)	103 (6.4%)	91 (56.5%)	44 (44.0%)	17 (47.2%)
Cancer – AIDS	2 (0.5%)	5 (3.6%)	0 (0%)	6 (3.7%)	1 (0.6%)	0 (0%)	0 (0%)
Cancer	23 (6.4%)	11 (8.0%)	0 (0%)	4 (2.5%)	9 (5.5%)	17 (17.0%)	4 (11.1%)
Accidents or violent death	20 (5.6%)	1 (0.7%)	0 (0%)	8 (5.0%)	10 (6.2%)	3 (3.0%)	0 (0%)
Other cardiovascular causes	7 (1.9%)	2 (1.4%)	0 (0%)	0 (0%)	5 (3.1%)	2 (2.0%)	2 (5.5%)
Definitive acute myocardial infarction	6 (1.6%)	1 (0.7%)	0 (0%)	1 (0.6%)	4 (2.4%)	2(2.0%)	0 (0%)
Other causes of the central nervous system	2 (0.5%)	2 (1.4%)	0 (0%)	1 (0.6%)	2 (1.2%)	0 (0%)	1 (2.7%)
Other urogenital diseases	5 (1.4%)	1 (0.7%)	0 (0%)	0 (0%)	2 (1.2%)	3 (3.0%)	1 (2.7%)
Bacterial with sepsis	2 (0.5%)	2 (1.4%)	0 (0%)	0 (0%)	1 (0.6%)	2 (2.0%)	1 (2.7%)
Unknown with sepsis	2 (0.5%)	0 (0%)	0 (0%)	0 (0%)	0 (0%)	2 (2.0%)	0 (0%)
Chronic obstructive pulmonary disease	1 (0.2%)	0 (0%)	0 (0%)	0 (0%)	0 (0%)	0 (0%)	1 (2.7%)
Pulmonary embolism	1 (0.2%)	0 (0%)	0 (0%)	0 (0%)	0 (0%)	1 (1.0%)	0 (0%)
Other causes of the digestive system	4 (1.1%)	1 (0.7%)	0 (0%)	0 (0%)	2 (1.2%)	2 (2.0%)	1 (2.7%)
Liver failure	2 (0.5%)	0 (0%)	0 (0%)	0 (0%)	2 (1.2%)	0 (0%)	0 (0%)
Hepatitis B	1 (0.2%)	0 (0%)	0 (0%)	0 (0%)	0 (0%)	1 (1.0%)	0 (0%)
Gastrointestinal bleeding	0 (0%)	1 (0.7%)	0 (0%)	0 (0%)	1 (0.6%)	0 (0%)	0 (0%)
Diabetes mellitus	1 (0.2%)	0 (0%)	0 (0%)	0 (0%)	0 (0%)	1 (1.0%)	0 (0%)
Bacterial	1 (0.2%)	0 (0%)	0 (0%)	0 (0%)	1 (0.6%)	0 (0%)	0 (0%)
Chronic alcoholism	5 (1.4%)	2 (1.4%)	0 (0%)	1 (0.6%)	3 (1.8%)	1 (1.0%)	2 (5.5%)
Non-classified causes	18 (5.0%)	7 (5.1%)	2 (5.5%)	7 (4.4%)	6 (3.7%)	7 (7.0%)	3 (8.3%)
Other causes	3 (0.8%)	2 (1.4%)	0 (0%)	3 (1.8%)	1 (0.6%)	1 (1.0%)	0 (0%)
Unknown causes	8 (2.2%)	2 (1.4%)	1 (2.7%)	4 (2.5%)	4 (2.4%)	1 (1.0%)	0 (0%)

**Table 5:** Distribution by group of the underlying cause of death of people living with HIV/AIDS according to sex and age group.

	Male (n = 356)	Female (n = 136)	20 to 29 (n = 36)	30 to 39 (n = 159)	40 to 49 (n = 161)	50 to 59 (n = 100)	60 + (n = 36)
**Underlying cause per group of diseases**							
Group 1 – AIDS	**259(72.7%)**	**103 (75.7%)**	**35(97.2%)**	**131(82.3%)**	**113 (70.1%)**	**60(60.0%)**	**23 (63.8%)**
Group 2 – Cancer AIDS	2 (0.5%)	5 (3.6%)	0 (0%)	6 (3.7%)	1 (0.6%)	0 (0%)	0 (0%)
Group 3 – Cancer	23 (6.4%)	11 (8.0%)	0 (0%)	4 (2.5%)	9 (5.5%)	17 (17.%)	4(11.1%)
Group 4 – External causes (Accident/violence)	28 (7.8%)	3 (2.2%)	1 (2.7%)	12 (7.5%)	14 (8.6%)	4 (4.0%)	0 (0%)
Group 5 – Cardiovascular diseases	13 (3.6%)	3 (2.2%)	0 (0%)	1 (0.6%)	9 (5.5%)	4 (4.0%)	2(5.5%)
Group 6 – Nervous system diseases	2 (0.5%)	2 (1.4%)	0 (0%)	1 (0.6%)	2 (1.2%)	0 (0%)	1 (2.7%)
Group 7 – Genitourinary disorders	5 (1.4%)	1 (0.7%)	0 (0%)	0 (0%)	2 (1.2%)	3 (3.0%)	1 (2.7%)
Group 8 – Respiratory diseases	7 (1.9%)	1 (0.7%)	0 (0%)	0(0%)	0 (0%)	6 (6.0%)	2 (5.5%)
Group 9 – Liver diseases	7 (1.9%)	1 (0.7%)	0 (0%)	1 (0.6%)	4 (2.4%)	1 (1.0%)	2 (5.5%)
Group 10 – Digestive system diseases	3 (0.8%)	1 (0.7%)	0 (0%)	0 (0%)	1 (0.6%)	2 (2.0%)	1 (2.7%)
Group 11 – Other causes	7 (1.9%)	5 (3.6%)	0 (0%)	3 (1.8%)	6(3.7%)	3 (3.0%)	0 (0%)

## DISCUSSION

The results of this study have demonstrated an annual mortality rate of 1.46 deaths per 100 PLHIV (95% CI: 1.33 – 1.60). The probability of death in the first year was 1.3%, which increased to 5.6% in five years, and to 26.7% in 20 years. Despite the natural increase in the cumulative probability of death, the survival curve demonstrated linearity, revealing slight changes in the probability of death over time. Mortality studies among PLHIV have presented variations in the mortality rates. A cohort conducted in the USA^11^ from 1995 to 2015 with 1,645 participants, presented an annual mortality rate of 1.19 deaths per 100 PLHIV, whereas another cohort in Southeast Asia^12^ from 2012 to 2016, including 1,990 patients reported an annual mortality rate of 2.16 per 100 PLHIV. A cohort conducted in East Asia^13^ with 33,142 persons living with HIV, during the same period, found 1.90 deaths per 100 PLHIV per year. These data indicate that Brazil presents an intermediate annual mortality rate, placed between the rates reported in the US and Asia, with discernibly better results when compared to Southeast Asia, a region with a large number of HIV cases in low- to middle-income countries with recent and unsystematic urbanization, which has resulted in difficulties in accessing antiretroviral therapy and health care^11-13^. This reinforces Joint United Nations Program on HIV/AIDS (UNAIDS) data, which demonstrate that socioeconomic contexts and structural measures are involved in the incidence of new infections and mortality in the population living with HIV^1^ .

Comparing the survival curves according to sex, a 49% higher risk of death was observed for males. This result corroborates the data reported by Santos *et al*.^14^ in a study including 411,028 patients followed up from 2007 to 2015 in Brazil, Dovel *et al*.^15^ in Africa, and Croxford *et al*.^16^ in Europe, from 1997 to 2012, with 448,839 person-years of follow-up, who all reported higher rates of all-cause mortality among males in PLHIV. A meta-analysis including 108 studies demonstrated that men living with HIV have higher risks of all-cause mortality compared to women, with persistent disparities in mortality between sexes over treatment^17^. This increase in the risk of death for men compared to women may be explained by the fact that men initiate ART in more advanced stages of the disease and present lower adherence to treatment and greater loss to follow-up on ART, as shown in a study conducted from 2004 to 2009 in South Africa with 1,154 participants^18^, in addition to a higher all-cause mortality rate when compared to women. These data reinforce the need for investment in programs that provide guidance on the importance of treatment and health care for PLHIV, with particular focus on the male population. The creation of specific services that facilitate the access of men into the health system and allow an early HIV diagnosis and self-testing are strategies recommended by UNAIDS to reduce these disparities^1^.

Regarding age, there was a difference between the survival curves, highlighting higher mortality among those aged 50 years and over at the time of HIV diagnosis, with a significant increase of 51% on the risk of death, which was also observed in the study by Santos *et al.* ^14^, in which mortality in people aged 50 years and over almost doubled when compared with those aged 18–39 years. Similar results were also presented in studies by Mangal *et al*.^19^, conducted in Brazil from 2006 to 2015 including 269,076 individuals, and by Carriquiry *et al.* ^20^, conducted in Latin America and the Caribbean from 2000 to 2014 with 16,996 patients. A French study involving 1,415 PLHIV, conducted from 2008 to 2012, showed that higher mortality rates may be related to the greater occurrence of chronic degenerative diseases in the population aged 50 years and over, as well as greater occurrence of chronic inflammation caused by HIV and longer period of exposure to ART^21^.

At the time of data collection, despite 85.7% respondents undergoing ART, the causes of death related to AIDS presented a higher percentage in the study (73.5%), whereby younger patients (20 to 29 years) presented with 97.2% of AIDS-related causes of death. In line with our results, a study by Croxford *et al*.^16^ also reported AIDS-related conditions as the main cause of death. In a survey conducted in Southeastern Brazil from 1999 to 2015, AIDS-related diseases were found to be the main cause of death^22^. A study on adherence to treatment^23^ conducted in the same state of Brazil, conducted from 2012 to 2013 with 253 patients, reported a non-adherence of 28.4%, with statistical associations between non-adherence to ART and younger individuals. Individuals who experienced greater difficulties in consulting their physicians and those who reported lack of medication presented higher rates of abandonment. In contrast to this, adherent individuals reported obtaining more information about ART from their physicians, which could be associated with better treatment. Our data and those presented by the adherence study suggest that difficulties may still be observed regarding adherence to treatment in the population living with HIV in the studied region, especially among younger people. This reinforces the importance of health services in relation to the management of adherence to treatment in order to reduce the incidence of AIDS-related opportunistic diseases and, consequently mortality.

The second major cause of death in the study was non-AIDS-related cancer, with an increased frequency of death with increasing age, found mainly in the age groups over 40 years. A study conducted from 1999 to 2011, involving 11 large cohorts and 49,731 participants, carried out in Europe, the USA, and Australia^6^, also reported cancer as the most common cause of non-AIDS-related deaths. Corroborating our findings, in a study conducted with 2,797 PLHIV in Japan from 2005 to 2016, Nishijima *et al*.^24^ reported a higher percentage of deaths from non-AIDS-defining malignancies in the population aged from 36 to 55 years, inferring that an older age may be associated with the occurrence of cancer. The reasons for the high rate of non-AIDS-related cancer in this population are unclear, although PLHIV present a high prevalence of cancer risk factors, such as smoking habit, alcohol use, chronic viral hepatitis infection, and other oncogenic viruses such as human papilloma virus. In addition, greater longevity after the global implementation of ART may be related to the development of cancer since the incidence of most cancers increases with age^25^. A study conducted in the USA from 1996 to 2010, with 3,045 HIV-infected patients with cancer and 1,087,648 patients with cancer and without HIV infection, showed that other factors that may contribute to deaths from cancer would be difficulty in treatment due to the aggressiveness of the cancer caused by immunosuppression, drug interaction between antineoplastic and antiretroviral drugs, the direct role played by HIV in carcinogenesis, inhibiting tumor suppressor genes and cellular proto-oncogenes, and a lack of healthcare services for managing treatment for both conditions concomitantly^26^.

The differences in cancer mortality rate according to sex remains unclear. Our study presented higher mortality rates among women, thereby corroborating the findings of Croxford *et al.* ^16^. However, this predominance varied according to the type of cancer in two other studies^27,28^. Nonetheless, increasingly high rates of cancer mortality are observed in women with HIV, especially anal, breast, ovarian, uterine, and lung cancers^27^. Overall, the probability of developing cancer throughout life is higher for men, although some cancers are more common in women^29^. It should be considered that a higher proportion of men die young, mainly due to AIDS-related causes, a fact already demonstrated in this study, while women with HIV tend to live longer^19^. Thus, further studies are suggested to better clarify the development of cancer and death from this cause in people living with HIV, according to sex.

Cardiovascular diseases were also prominent in the study among the main causes of non-AIDS-related death (3.2%), in agreement with results found in Brazil by Grinsztejn *et al*.^30^, including 3,530 patients in metropolitan areas of Rio de Janeiro State from 1986 to 2009. People living with HIV present a high risk for the occurrence of cardiovascular disease for which there are several associated factors, such as the interaction between traditional risk factors, HIV-specific factors such as chronic inflammation and immune activation, ART-related dyslipidemia, behavioral factors, and difficulties in accessing health care^31^. Thus, the importance of prevention and control of these associated factors is reinforced, in addition to the effective treatment of cardiovascular diseases in this population.

Death from external causes (accident/violent death) appeared as the third leading cause of non-AIDS-related deaths, thereby drawing attention to a higher percentage of death from this cause in those aged from 30 to 49 years. Studies in Brazil^8,22^ and a large population-based study in the UK^17^ also reported external causes among the main causes of death, also finding an increase in the number of deaths from this cause over time. This high rate is probably associated with behavioral factors in this population, such as greater exposure to illicit drug use and alcohol consumption, which are related to the occurrence of violence and accidents, as evidenced in an Australian study conducted from 1999 to 2012 and a review study conducted by McManus *et al*.^32^ and Petoumenos *et al*.^33^. These habits are also related to a lower life expectancy, an aspect that may have occurred in our present study, in which deaths from external causes were more predominant among those aged 50 years and under^34^.

### Strengths and limitations of the study

This study presents the mortality profile in the population living with HIV over a long period of follow-up, highlighting important causes of death in addition to AIDS-related diseases, such as chronic degenerative diseases, cancer, and cardiovascular diseases. These data highlight the need for care centers with multiple specialties and easy access to track these diseases throughout the follow-up of these patients. A more complete database on patient characteristics, such as socioeconomic profile; habits, such as smoking, alcohol consumption, and drug use; and conditions, such as diabetes mellitus, hypertension, and chronic kidney disease would allow for a better discussion on the mortality profile found in this population. Furthermore, The ICD-10 death classification code described in the research database informed the underlying cause of death, which may justify the non-appearance of some comorbidities such as Hepatitis C.

## CONCLUSION

This study demonstrated that AIDS-related diseases remain the main cause of death, suggesting the need for better strategies to ensure that patients maintain their links to the health service and adherence to treatment. Accident/violent deaths were the third leading cause of death, highlighting the need for improving access to interventions, such as drug and alcohol rehabilitation and mental health services. Moreover, it also underlines the need for permanent vigilance by healthcare professionals on health risks faced by PLHIV. Chronic and degenerative diseases, such as cancer and cardiovascular diseases, are significant causes of death, indicating that tracking these diseases throughout the follow-up period is essential, as well as following specific treatment guidelines and facilitating access to care centers with multiple specialties. Infection diagnosis at an older age and the male sex were also found to be risk factors for higher mortality rates, emphasizing the need for greater caution in treating the infection and managing comorbidities in these specific groups.
